# Cardioprotective aspirin users and their excess risk of upper gastrointestinal complications

**DOI:** 10.1186/1741-7015-4-22

**Published:** 2006-09-20

**Authors:** Sonia Hernández-Díaz, Luis A García Rodríguez

**Affiliations:** 1 Department of Epidemiology, Harvard School of Public Health, Boston, MA, USA; 2 Centro Español de Investigación Farmacoepidemiológica (CEIFE), Madrid, Spain

## Abstract

**Background:**

To balance the cardiovascular benefits from low-dose aspirin against the gastrointestinal harm caused, studies have considered the coronary heart disease risk for each individual but not their gastrointestinal risk profile. We characterized the gastrointestinal risk profile of low-dose aspirin users in real clinical practice, and estimated the excess risk of upper gastrointestinal complications attributable to aspirin among patients with different gastrointestinal risk profiles.

**Methods:**

To characterize aspirin users in terms of major gastrointestinal risk factors (i.e., advanced age, male sex, prior ulcer history and use of non-steroidal anti-inflammatory drugs), we used The General Practice Research Database in the United Kingdom and the Base de Datos para la Investigación Farmacoepidemiológica en Atención Primaria in Spain. To estimate the baseline risk of upper gastrointestinal complications according to major gastrointestinal risk factors and the excess risk attributable to aspirin within levels of these factors, we used previously published meta-analyses on both absolute and relative risks of upper gastrointestinal complications.

**Results:**

Over 60% of aspirin users are above 60 years of age, 4 to 6% have a recent history of peptic ulcers and over 13% use other non-steroidal anti-inflammatory drugs. The estimated average excess risk of upper gastrointestinal complications attributable to aspirin is around 5 extra cases per 1,000 aspirin users per year. However, the excess risk varies in parallel to the underlying gastrointestinal risk and might be above 10 extra cases per 1,000 person-years in over 10% of aspirin users.

**Conclusion:**

In addition to the cardiovascular risk, the underlying gastrointestinal risk factors have to be considered when balancing harms and benefits of aspirin use for an individual patient. The gastrointestinal harms may offset the cardiovascular benefits in certain groups of patients where the gastrointestinal risk is high and the cardiovascular risk is low.

## Background

Cardiovascular disease is the leading cause of morbidity and mortality in Europe and North America [1]. The overall incidence of myocardial infarction is around 1 to 3 cases per 1,000 person-years [1,2]. However, a given person's risk for myocardial infarction varies significantly with a number of factors including age, sex, blood pressure, cigarette smoking, diabetes, cholesterol levels, and a previous diagnosis of cardiovascular disease [3]. This risk variability is so widely accepted that the specific cardiovascular risk for a given person can be formally estimated using equations currently available on the web [4].

Long-term use of low-dose aspirin reduces the risk of myocardial infarction by about 25%–30% irrespective of the baseline risk [5-7], therefore this relative change has a greater absolute impact on persons with an elevated underlying risk. It has been estimated that treating patients with a moderately high baseline risk with aspirin would prevent up to 4 myocardial infarctions per 1,000 persons treated per year, while treating low risk patients would prevent less than 1 event per 1,000 persons treated per year [8].

The cardiovascular benefits from low-dose aspirin have to be balanced against the potential gastrointestinal harm. Observational studies have estimated that the incidence of serious upper gastrointestinal tract complications (UGIC; i.e., bleeding or perforation) in the general population is on the order of 1 case per 1,000 person-years, with a case fatality rate around 5–10% [9]. Aspirin use, even at doses below 300 mg/day and despite modified release formulations, has been associated with a two to threefold increased risk of developing UGIC [10]. Based on these data, the average incidence of UGIC would be close to 2–3 cases per 1,000 person-years for aspirin users, and about 1–2 of these cases might be attributable to aspirin. Clinical trials, typically conducted in low-risk subjects, have also estimated an overall excess of 1–2 cases of UGIC per 1,000 patients treated with aspirin per year [8].

After considering the absolute increase in bleeding complications associated with aspirin, several studies have suggested that aspirin use for prevention of cardiovascular events might be harmful for groups of patients at low risk for coronary heart disease (< 5 events per 1,000 person-years) and clearly beneficial for high-risk groups (≥15 events per 1,000 person-years) [3,11,12]. While these studies considered various cardiovascular risk factors to determine the coronary heart disease risk for each individual, they did not take into account the independent gastrointestinal risk factors to determine individualized UGIC risks.

Estimates of the specific UGIC risk for each person would add a valuable piece of information to individual therapeutic decisions and could optimize the overall risk-benefit profile of aspirin. Therefore, we describe below i) the distribution of the main risk factors for UGIC among populations of prescription aspirin users, ii) the estimated baseline risk of UGIC within levels of these factors, and iii) the estimated excess risk of UGIC that could be attributed to aspirin within subgroups of aspirin users with specific baseline risks of UGIC.

## Methods

### Distribution of major risk factors

To describe the distribution of major gastrointestinal risk factors among aspirin users, we used two different sources of data:

#### The General Practice Research Database (GPRD)

The GPRD is a population-based database in the United Kingdom where general practitioners retrieve and store on computer clinical information on their patients, including demographics, diagnoses and comments, referral information and records of all prescriptions issued by them, as well as their dose and indication [13]. All these data are automatically and continuously passed on to the GPRD. Data on about 3 million patients are systematically recorded and sent anonymously to the Medicines and Healthcare products Regulatory Agency. This agency collects and organizes this information in order for it to be used for research projects. The Read classification system is used to code specific diagnoses, and a drug dictionary based on data from the Prescription Pricing Authority is used to code drugs. The accuracy and completeness of these data have been validated in previous studies [14,15].

The source population for the current study encompassed a cohort of patients aged 20 to 99 years in the year 2000 who had been enrolled for at least two years with the GP. For each patient, we selected a random date during 2000 as the index date and considered this date as a snapshot of the population. We defined patients as "aspirin users" when the supply of a prescription for aspirin lasted until the index date or ended within the period of 3 months before index date (N = 16,598). Within aspirin users, the following characteristics were studied as important risk factors for UGIC: history of upper gastrointestinal disorder while enrolled in the database (dyspepsia, peptic ulcer, upper gastrointestinal bleeding/perforation), sex, age, and current or recent use of non-aspirin non-steroidal anti-inflammatory drugs (NSAIDs) by the index date (i.e., prescription lasted until the index date or ended within the 3 months before).

#### Base de Datos para la Investigación Farmacoepidemiológica en Atención Primaria (BIFAP)

BIFAP is a population-based database in Spain where general practitioners retrieve and store on computer clinical information on their patients including demographics, diagnoses and comments, referral information and records of all prescriptions issued by them [16]. All this information is automatically and periodically passed on to BIFAP. Data on an excess of 1 million patients is systematically recorded and sent anonymously to the Spanish Medicines Agency. This agency collects and organizes the information. The ICPC classification system is used to code specific diagnoses, and the National Dictionary of Drugs is used to code drugs. This database is currently in its pilot phase and the accuracy and completeness of the data source is being examined [17].

The source population for the current study encompassed a cohort of patients aged 20 to 99 years in the year 2000. We selected a random date during 2000 as the index date and consider this date as a snapshot of the population. We defined patients as "aspirin users" when the supply of a prescription for aspirin lasted until the index date or ended within the period of 3 months before index date (N = 9,129). We classified aspirin users according to the indication for aspirin. Within patients treated with aspirin for cardioprotection, the following characteristics were studied as important risk factors for UGIC: history of upper gastrointestinal disorder, sex, age, and current or recent use of NSAIDs by the index date (i.e., prescription lasted until the index date or ended within the 3 months before).

### Specific risks and excess risks

To estimate the baseline incidence rate of UGIC according to major gastrointestinal risk factors and the excess risk of UGIC that could be attributed to aspirin within levels of these factors, we analyzed data from the GPRD and conducted systematic reviews of the literature.

#### The General Practice Research Database (GPRD)

Original analyses from the GPRD have been already published [18,19]. Briefly, the source population encompassed patients aged 40 to 79 years between April 1993 to October 1998, with at least 2 years' enrollment in the database. We identified incident cases of UGIC that were referred to a specialist or admitted to a hospital. Controls frequency-matched for age, sex and calendar year were selected randomly from the person-time at risk of the source population. The index date was the date of the first diagnosis for cases and a random date for controls. We defined patients as "current users" of aspirin or NSAIDs when the supply of a prescription lasted until the index date or ended within a window of 30 days before the index date. Non-users were those with no prescription ever recorded before the index date. Adjusted estimates of relative risk associated with the current use of aspirin and NSAIDs compared to non-use, as well as the effects and the potential interaction with other risk factors were studied using unconditional logistic regression.

#### Systematic reviews of the literature

We have conducted and published systematic reviews on both absolute and relative risks of UGIC, which included our own estimates from the GPRD [9,10,20]. The studies included in these meta-analyses defined UGIC as bleeding, perforation, or other serious upper gastrointestinal tract event resulting in hospitalization or visit to a specialist. The literature suggests that the major risk factors for the development of UGIC are being aged over 60, male gender, history of peptic ulcer, and concomitant use of NSAIDs. All the estimates mentioned below are based on pooled relative risks (RR) from meta-analyses and have very tight confidence intervals. For example, the pooled RR was 2.5 (95% CI: 2.4, 2.7) for aspirin overall and 2.0 (95% CI: 1.7–2.2) for doses up to 300 mg/day.

#### Risk, relative risk, and excess risk estimates

To estimate absolute incidence rates within each risk subgroup, and based on pooled estimates from ours and others' original studies, [9,10,20] we assumed the following: i) The overall baseline UGIC incidence rate is in the order of 1 per 1,000 person-years (pooled incidence rate = 0.99; 95%CI 0.84–1.18). ii) The incidence increases exponentially with age, from less than 1 per 1,000 person-years until the age of 60 to over 5 per 1,000 person-years at age 85 years. iii) Within age groups, the incidence rates among men are double those among women. iv) Regarding previous ulcer history, the relative risks used for past upper gastrointestinal pain/dyspepsia, uncomplicated and complicated ulcers were 2, 6, and 10, respectively. v) The relative risk associated with NSAIDs has been shown to be lower among patients with past ulcer history, even though the absolute risk is still higher among NSAID users with ulcer history. Briefly, exposure to NSAIDs causes over a fourfold increase in the risk of UGIC for patients without prior history (from 1 to 4 cases per 1,000 persons per year), and a greater than twofold increase in patients with a history of peptic ulcer disease (from 10 to 25 cases per 1,000 persons per year). Hence, the effect of NSAIDs on the risk of UGIC decreases proportionally with the severity of the prior gastrointestinal condition. However, patients with a history of complicated ulcer disease present the greatest absolute risk of UGIC when taking NSAIDs, that is, about 25 cases per 1,000 person-years, from which 15 cases could be attributable to NSAID treatment. The relative risks used for NSAID use were 2.5 in subjects with a past history of complicated or uncomplicated ulcer, 3 in subjects with past upper gastrointestinal pain, and 4 in subjects with no previous upper gastrointestinal complaint. vi) Low-dose aspirin doubles the risk of UGIC in each of these risk groups.

The difference in UGIC rates between aspirin users and non-users within levels of major risk factors was considered as excess risk attributable to aspirin use (i.e., we assumed no residual confounding). To evaluate the sensitivity of the results to our assumptions, we repeated the analyses for a range of plausible values for each estimate. The sensitivity analysis for the effect of aspirin on UGIC, for which RRs of 1.5, 2, and 3 were considered, is presented here. To validate our calculations, we applied our UGIC risk estimations to the populations of patients included in the control groups of recent clinical trials [21-23], and compared our estimates with the UGIC risks reported in these studies.

## Results

**Figure 1:**
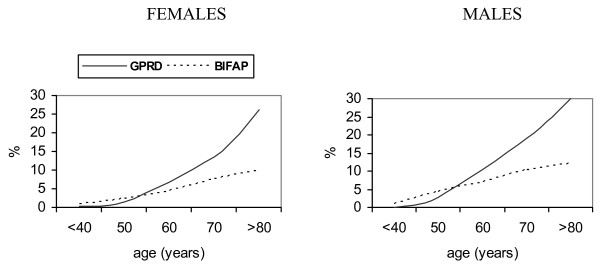
Prevalence of aspirin use for cardioprotection in the UK (General Practice Research Database; GPRD) and Spain (Base de Datos para la Investigación Farmacoepidemiológica en Atención Primaria; BIFAP) by age for females and males.

The prevalence of prescription aspirin use was 4.6% in the GPRD and 3.4% in BIFAP. Over 95% of the aspirin use recorded in the GPRD and 59% of the use in BIFAP was for cardioprotection. In both data sources the prevalence of aspirin use increased substantially with age (Figure 1).

**Figure 2:**
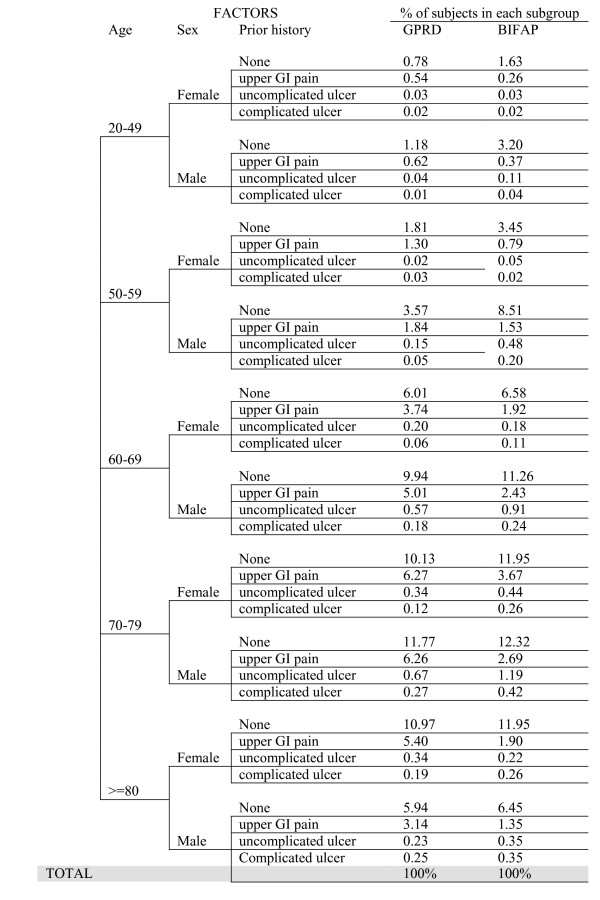
Distribution of risk factors for upper gastrointestinal tract complications (UGIC) among cardioprotective aspirin users in the UK (General Practice Research Database; GPRD) and Spain (Base de Datos para la Investigación Farmacoepidemiológica en Atención Primaria; BIFAP).

The distribution of gastrointestinal risk factors among cardioprotection aspirin users is presented in Figure 2. Overall, the proportion of aspirin users above 60 years of age was 88% in the GPRD and 79% in BIFAP. The proportion of males was 52% in the GPRD and 54% in BIFAP. The proportion of aspirin users with a recorded history of gastrointestinal ulcer was 3.8% in the GPRD and 5.9% in BIFAP. The proportion of aspirin users with a recorded past episode of UGIC was 1.2% in the GPRD and 1.9% in BIFAP. The proportion of aspirin users concomitantly using NSAIDs was 14.8% in the GPRD and 13.2% in BIFAP. NSAID use was not included in the figure to simplify the presentation.

**Figure 3:**
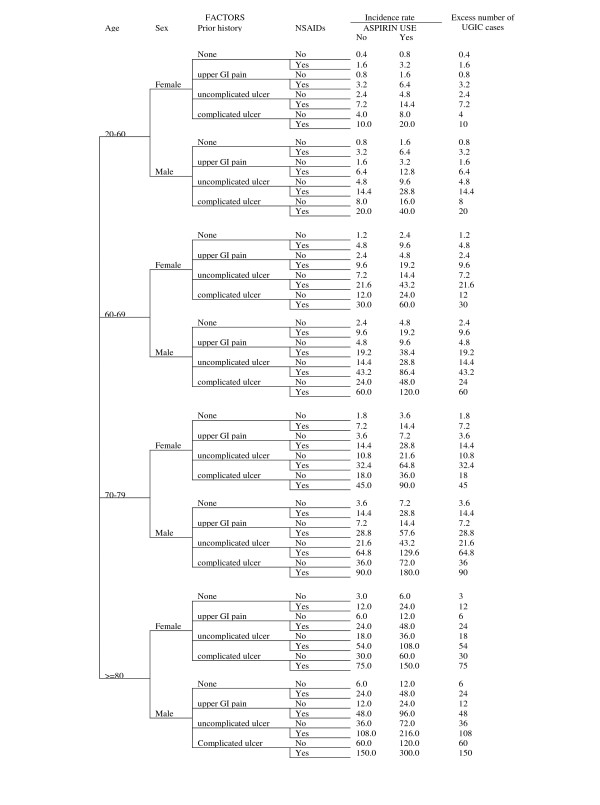
Estimated incidence rate of upper gastrointestinal tract complications (UGIC) per 1,000 person-years and estimated excess number of cases per 1,000 cardioprotection aspirin users per year attributable to aspirin within levels of gastrointestinal risk factors.

Figure 3 depicts how the estimated incidence rate of UGIC increases with age and with past history of ulcer both for males and females. These rates would be around four times higher among NSAID users and would double with aspirin use. Taking into account the distribution of baseline gastrointestinal risk factors among aspirin users in each population, the estimated average incidence rate of UGIC among aspirin users was 12 cases per 1,000 person-years in the GPRD and 10 in the BIFAP. However, this rate can be more than 20 cases per 1,000 person-years in certain high-risk groups. For example, considering, age, sex, ulcer history and use of NSAIDs as stratifying factors, the expected UGIC incidence rate would be above 20 per 1,000 person-years in 14% and 11% of aspirin users, based on GPRD and BIFAP, respectively. Moreover, in a smaller fraction of the population, for example males 70 and older with a recent history of peptic ulcer and who take aspirin and NSAIDs concomitantly, the risk might exceed 100 cases per 1,000 person-years.

The number of cases that could be attributable to aspirin among aspirin users according to age, sex, and ulcer history is also presented in Figure 3. For cardioprotection aspirin users overall, the estimated excess risk of UGIC was 6 and 5 cases per 1,000 users per year in the GPRD and BIFAP, respectively. These figures would be larger in high-risk groups. For example, patients with baseline incidence rates over 20 per 1,000 person-years, such as males 70 years and older with a past history of uncomplicated peptic ulcer, would develop over 40 cases per 1,000 person-years if they were treated with low-dose aspirin; thus, more than 20 cases per 1,000 aspirin users with these risk factors per year could be attributable to their use of aspirin.

**Figure 4:**
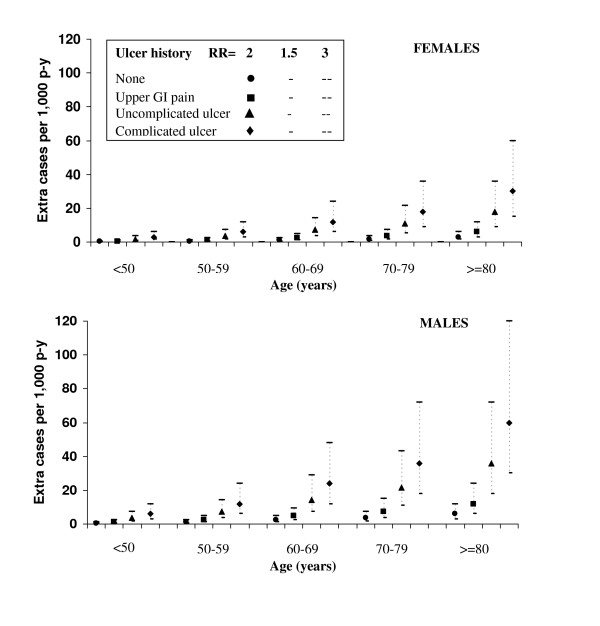
Sensitivity analysis. Estimated excess number of upper gastrointestinal tract complications (UGIC) cases attributable to aspirin (measured as the incidence rate difference between users and non-users) stratified by prior ulcer history and age for male and females, assuming a range of relative risks for aspirin from 1.5 to 3.

The number of extra cases attributable to aspirin depends on the assumptions regarding incidence rates, distribution of risk factors, and relative risks used throughout the analyses. Estimates were particularly sensitive to the value used for the relative risk of UGIC associated with aspirin use. Figure 4 presents the number of extra cases potentially attributable to aspirin according to age, sex, and ulcer history for a plausible range of relative risks associated with low-dose aspirin (i.e., from 1.5 to 3).

Based on our estimates, the expected incidence of UGIC in the control groups of the Vioxx Gastrointestinal Outcomes Research (VIGOR) trial [21], the Celecoxib Long-term Arthritis Safety Study (CLASS) [22], and the Therapeutic Arthritis Research and Gastrointestinal Event Trial (TARGET) [23] would have been 7, 9.5 and 8.1 cases per 1,000 person-years, respectively; the actual reported annualized rates were 8.6, 14.5, and 9.1 per 1,000 person-years, respectively (Table 1).

## Discussion

The prevalence of cardioprotective aspirin use among adults in year 2000 was 4.6% in the UK and 2.0% in Spain according to European databases. The prevalence increased substantially with age and was slightly higher in men than in women, so that use was above 10% among men over 65 years of age. These data agree well with other studies from the UK and Spain that reported a prevalence of regular aspirin use of 8–9% in elderly populations [24,25].

In the US, according to the population-based National Health and Nutrition Examination Survey (NHANES) [26], close to 9% of adults were using an aspirin containing product regularly in the 2000–2002 period, although the prevalence of aspirin use was above 30% in men over 65 years of age. Similarly, a contemporaneous US survey specifically designed to provide information on drug use reported a prevalence of aspirin use that ranged from 10% in women and men aged 18–44 years of age to 39% in men over 65 [27]. The higher prevalence of aspirin use in the US surveys might be due not only to actual differences in aspirin use or aspirin users in the US and Europe, but also to methodological aspects. For example, the European databases used for the current study included only prescription aspirin, focused on single component products used for cardioprotection and were based on prospectively recorded prescriptions; while the US surveys included prescription and nonprescription aspirin used regularly for any indication and both as single component or as part of multiple component products, and were based on subject recall. Of note, the lack of information on over the counter aspirin use would have a limited impact on this particular study as we were interested in users of aspirin for cardioprotection, and i) most regular aspirin users in the UK and Spain seek prescriptions in order to benefit from the drug coverage offered by the universal medical care, and ii) even if we had missed a fraction of cardioprotective aspirin users, those included in our population would probably be representative in terms of UGIC risks and, therefore, valid for the objectives of the study.

The specific UGIC risk for a given person according to the most relevant gastrointestinal risk factors can be calculated using the estimates provided in this paper. In addition, these estimates can be used to determine the expected number of UGIC cases in a particular group of individuals during a given period of time. For example, our estimates of the incidence of UGIC in the control groups of VIGOR [21], CLASS [22], and TARGET [23] trials were strikingly close to the observed ones, despite the distinct methodological characteristics of the trials.

This paper also provides estimates for the absolute impact of aspirin treatment on persons with different underlying UGIC risk profiles. The excess risk attributable to aspirin in the general population has traditionally been suggested to be around 1–2 cases per 1,000 patients treated with low-dose aspirin per year [8]. However, this figure largely applies to a middle-aged group of people with no prior history of peptic ulcer and may substantially underestimate the bleeding risk among aspirin users in a real-life setting, where persons over 65 are precisely the group most likely to be taking low-dose aspirin for its cardioprotective properties. We estimated an overall excess risk of 5 cases per 1,000 aspirin users per year. Yet this is an average figure; the excess risk varied in parallel to the baseline UGIC incidence rate and ranged from less than 1 case to over 10 extra cases per 1,000 aspirin users per year for patients with low or high underlying gastrointestinal risk, respectively.

Therefore, age, together with other major risk factors for UGIC such as prior history of gastrointestinal ulcer, male gender, or concomitant use of NSAIDs, should be considered when balancing the risks and benefits of low-dose aspirin for a particular subgroup of patients. Estimates of the absolute impact of aspirin treatment can assist patients and health care providers in evaluating the magnitude of the trade-offs involved in taking aspirin for a particular individual. Nonetheless, the decision making process will still face the challenge of weighing the benefits against the potential harm. That is, the number of cardiovascular events prevented cannot be directly compared with the number of bleeding events caused, as they are associated with different degrees of disability and mortality. Moreover, each person might weigh these beneficial and harmful effects differently [11].

Some of the estimates for the distribution of gastrointestinal risk factors among aspirin users presented in this article were based on relatively small numbers and, therefore, there is a level of uncertainty around them. Regarding the effect estimates, although we assigned reliable relative risks to each gastrointestinal risk factor (i.e., they were based on pooled estimates with tied confidence intervals), we had to make assumptions (e.g. that the relative risk for aspirin is constant across certain groups with different baseline UGIC risks) that, while reasonable based on current knowledge, might be proven partly wrong in future studies. For simplicity, we restricted the number of gastrointestinal risk factors to those with greatest potential impact due to their high prevalence and/or relative risk; less frequent (e.g. use of anticoagulants, other comorbidities) and weaker (e.g. smoking or alcohol drinking) risk factors for UGIC were not considered. Also, while information on other risk factors such as *Helicobacter pylori *infection or genetic predisposition is rarely available at present, these and other markers will probably become important determinants of a person UGIC risk in the future. Similarly, we did not include other benefits of aspirin treatment, such as the secondary prevention of serious vascular events other than myocardial infarction [5,6] or the potential reduction in the risk of colon cancer [28] and cognitive decline in the elderly [29]; nor other potential areas of harm, such as the increased risk of hemorrhagic strokes [5,6] and uncomplicated peptic ulcers [30]. However, aspirin effects on the risks of myocardial infarction and UGIC are the most common, firmly established and serious and, therefore, would probably drive the decisions [12]. Finally, we did not discuss interventions that might modify the balance, such as use of a proton pump inhibitor.

## Conclusion

The net impact of aspirin on the risk of UGIC for a given person depends on the underlying gastrointestinal risk. The clinical decision about placing a person into low-dose aspirin for cardioprotection should consider not only the cardiovascular but also the gastrointestinal risk factors for each individual. This paper presents estimates of the UGIC risk for aspirin users with a wide range of gastrointestinal risk profiles.

## Competing interests

The authors have served as consultants for manufacturers of non-steroidal anti-inflammatory drugs. The Pharmacoepidemiology Program at the Harvard School of Public Health and CEIFE are partially supported by training and research grants from various pharmaceutical companies. The current manuscript was not directly financed by any organization.

## Authors' contributions

SHD participated in the design of the study, performed the statistical analysis and drafted the manuscript. LAGR conceived the study, participated in its design, and contributed to the interpretation of the results and writing of the manuscript. All authors read and approved the final manuscript.

## Pre-publication history

The pre-publication history for this paper can be accessed here:



## Figures and Tables

**Table 1:** Application of upper gastrointestinal tract complications  (UGIC) risk estimations to the populations of patients included in the control groups (exposed to traditional  non-steroidal anti-inflammatory drugs) for the Vioxx Gastrointestinal Outcomes Research (VIGOR), Celecoxib Long-term  Arthritis Safety Study (CLASS), and Therapeutic Arthritis Research and Gastrointestinal Event Trial (TARGET) trials

**Study**	**Age mean**	**Female %**	**Ulcer History %**	**Aspirin %**	**Reported Incidence Per 1,000 p-y**	**Estimated Incidence Per 1,000 p-y**
VIGOR [21]	58	80	7.8	0	8.6*	7.0
CLASS [22]	60	69	9.6	20	14.5	9.5
TARGET [23]	63	76	3.5	24	9.1	8.1

*Excluding 11 cases with gastrointestinal ulcer without apparent bleeding, obstruction, or perforation.
